# Encapsulation of Large-Size Plasmids in PLGA Nanoparticles for Gene Editing: Comparison of Three Different Synthesis Methods

**DOI:** 10.3390/nano11102723

**Published:** 2021-10-15

**Authors:** Tresa López-Royo, Víctor Sebastián, Laura Moreno-Martínez, Laura Uson, Cristina Yus, Teresa Alejo, Pilar Zaragoza, Rosario Osta, Manuel Arruebo, Raquel Manzano

**Affiliations:** 1 Department of Anatomy, Embryology and Animal Genetics, University of Zaragoza, Calle Miguel Servet 177, 50013 Zaragoza, Spain; 2 Centro de Investigación Biomédica en Red de Enfermedades Neurodegenerativas (CIBERNED), 28031 Madrid, Spain; 3 Agroalimentary Institute of Aragon (IA2), Calle Miguel Servet 177, 50013 Zaragoza, Spain; 4 Aragón Health Research Institute (IIS Aragón), 50009 Zaragoza, Spain; 5 Instituto de Nanociencia y Materiales de Aragón (INMA), CSIC-Universidad de Zaragoza, 50009 Zaragoza, Spain; 6 Department of Chemical Engineering, University of Zaragoza, Campus Río Ebro—Edificio I+D, C/Poeta Mariano Esquillor S/N, 50018 Zaragoza, Spain; 7 Networking Research Center on Bioengineering, Biomaterials and Nanomedicine, CIBER-BBN, 28029 Madrid, Spain

**Keywords:** plasmid-loaded nanoparticles, PLGA, biodegradable polymer, large-size plasmid, DNA, double emulsion solvent evaporation, batch-ultrasound, microfluidics, nanoprecipitation, gene therapy

## Abstract

The development of new gene-editing technologies has fostered the need for efficient and safe vectors capable of encapsulating large nucleic acids. In this work we evaluate the synthesis of large-size plasmid-loaded PLGA nanoparticles by double emulsion (considering batch ultrasound and microfluidics-assisted methodologies) and magnetic stirring-based nanoprecipitation synthesis methods. For this purpose, we characterized the nanoparticles and compared the results between the different synthesis processes in terms of encapsulation efficiency, morphology, particle size, polydispersity, zeta potential and structural integrity of loaded pDNA. Our results demonstrate particular sensibility of large pDNA for shear and mechanical stress degradation during double emulsion, the nanoprecipitation method being the only one that preserved plasmid integrity. However, plasmid-loaded PLGA nanoparticles synthesized by nanoprecipitation did not show cell expression in vitro, possibly due to the slow release profile observed in our experimental conditions. Strong electrostatic interactions between the large plasmid and the cationic PLGA used for this synthesis may underlie this release kinetics. Overall, none of the methods evaluated satisfied all the requirements for an efficient non-viral vector when applied to large-size plasmid encapsulation. Further optimization or alternative synthesis methods are thus in current need to adapt PLGA nanoparticles as delivery vectors for gene editing therapeutic technologies.

## 1. Introduction

In recent years, with the development of gene therapies, and especially the CRISPR/Cas system, the use of plasmids as therapeutic tools is emerging. Major drawbacks of this novel approach are the instability of nucleic acids, which are rapidly degraded by the endonucleases within the body [1], and the inefficient internalization of exogenous naked plasmids by cells. Furthermore, the components of the CRISPR/Cas system (the guide RNA and CRISPR-associated endonuclease) are often delivered into cells encoded on large-size plasmid vectors (9–19 kb) [2]. Due to their large size, these vectors are notably difficult to transfect and are potentially cytotoxic [3]. Therefore, the development of efficient and safe gene transfer vectors is essential for successful gene therapy.

Different gene delivery systems based on viral and non-viral vectors, such as lipoplexes or polymeric nanoparticles, have arisen [4,5,6,7], although none of them are so far fully satisfactory. Viruses provide high efficiency of transduction, persistence and cell tropism, but they have some important drawbacks that halt their implementation in clinical practice. These include the limited size of the cargo genetic material (packaging capacity), their random integration in the genome, the generation of an immunogenic response after repeated administrations and their potential toxicity [8]. Non-viral vectors are currently in the spotlight because of their advantages over viruses, such as their predictable safety profile, easy large-scale production and quality control, increased versatility, sustained cargo release and large-size nucleic acid loading capacity. However, their specificity and delivery efficacy are currently below those of viral systems.

In this sense, poly(lactide-co-glycolic acid) nanoparticles (PLGA NPs) have emerged as important vehicles in scientific and biomedical research fields thanks to their high biocompatibility and low toxicity [9]. One of the main advantages of PLGA is the generation of lactic and glycolic acids upon hydrolysis, two endogenous metabolites that can be degraded through the Krebs cycle [10]. This results in minimal toxicity and establishes PLGA as one of the most easily metabolized and tolerated polymers by the body. Moreover, PLGA NPs can encapsulate different cargos, from small hydrophobic and hydrophilic drugs to nucleic acids and proteins [11,12,13,14,15]. Furthermore, PLGA NPs can be easily conjugated to molecules modifying their charge, hydrophobicity, half-life in blood circulation and even allow selective binding to certain cell types [16].

These characteristics have potentiated the use of PLGA NPs as nano-drug delivery systems (nanoDDS) in a wide variety of diseases, including cardiovascular [17,18], neurodegenerative [19,20] and inflammatory and immune system diseases [21,22], infection [23], cancer [24,25], regenerative medicine [26,27] and even in the field of theragnostics and vaccines [28,29]. The efficacy of these PLGA-based nanoDDS has already been proven in clinical trials [30], and the manufacturing of drugs such as Atridox^®^ (to deliver doxycycline for periodontal treatment), Sandostatin^®^ (which contains octreotide for acromegaly) and Lupron depot^®^ (with leuprorelin for prostate cancer) has been approved by the FDA and the EMA and are already commercially available [31].

Unlike PLGA, nanoDDS-encapsulating drugs, the use of PLGA NPs as vectors for gene therapy is still in its infancy. In fact, with few exceptions, studies are restricted to in vitro assays and delivery of small non-coding RNAs such as siRNAs [32,33,34]. Moreover, it is noteworthy that most of the studies in which PLGA nanoparticles were used for plasmid encapsulation employed small-size constructs (below 6 kb) [35,36,37], while, as above mentioned, encoding gene editing components (i.e., Cas9 and guide RNAs) requires larger (9–19 kb) plasmids.

PLGA NPs can be synthesized using different techniques, thus achieving nanoparticles with differences in their properties in terms of size, hydrolysis time, shape, surface charge, cargo integrity and subsequent cell uptake efficiency.

The synthesis of PLGA nanoparticles through double water-in-oil-in-water (*w*/*o*/*w*) emulsion is the gold standard in the encapsulation of hydrophilic pharmaceutics, and derived modified methods have been developed depending on the specifications and needs of each application, since it confers the greatest achievable control over the physicochemical properties of the nanoparticles produced [35,38,39,40,41].

This technique consists of emulsifying, via sonication, a water-immiscible organic phase (oil) that contains the dissolved polymer that will form the nanoparticles with a surfactant or stabilizer (water). This first emulsion facilitates the formation of small polymer droplets. The resulting emulsion is added to a larger aqueous phase and stirred for several hours, allowing the solvent to evaporate and triggering nanoparticle formation [40]. Drugs can be dissolved into the organic phase if hydrophobic (when using single oil-in water emulsion) or into the first aqueous phase if hydrophilic (for the double *w*/*o*/*w* emulsion). In the case of PLGA-based nucleic acid-loaded nanoparticles to be used as gene therapy vectors, the double emulsion solvent evaporation method has been reported using different organic and aqueous solvents/surfactants such as polyvinyl alcohol (PVA), cetyltrimethylammonium bromide (CTAB), Span 80, sodium dodecyl sulphate (SDS), Triton X-100, Tween 20 and Tween 80 [35,42]. Nevertheless, emulsification by ultrasound sonication has several drawbacks: (1) limited scalability since it is only efficient for small batch preparations, (2) cross-contamination due to the debris produced in the sonotrode tip by cavitation and (3) the high voltage generates a high temperature that might influence temperature-sensitive cargoes [43].

Alternatively, nanoprecipitation (or the solvent displacement method) relies on the mixture of organic and aqueous miscible solvents, taking advantage of the polymer’s low solubility in one of those solvents to induce precipitation of the polymer into nanoparticles upon evaporation of the organic solvent, typically by stirring or by reduced pressure. Although this method was originally designed for hydrophobic compound entrapment [44], it has been recently modified for hydrophilic compounds, such as DNA [45,46]. 

Ultrasound-based emulsification and nanoprecipitation are mostly performed in discontinuous batch processes, where the reproducibility between batches and scalability is limited, hindering a fast transfer to the clinic. On the other hand, microfluidics is a novel technology that can be used to prepare polymer nanoparticles using single and double emulsion and nanoprecipitation, depending on the requirements, with the substantial advantage of continuous manufacturing and strict control on the synthesis parameters. Microfluidics allows exploiting the interfacial tension between two phases as the driving force for nanoparticle formation by molecular diffusion using microchannels integrated into microreactors [47]. The geometry of these microchannels (i.e., T-junctions, cross-junctions, Y-junctions and flow-focusing devices) enables multiple contact orientations, surfaces and flow rates between phases, resulting in nanoparticles with different properties [48]. Continuous flow microreactors allow greater control over the synthesis process, which is translated into reduced polydispersity and facilitates multi-stage processing [49]. This technique is relatively new and to the best of our knowledge, there is only one report on microfluidic-based synthesis of nucleic acid-loaded PLGA NPs which used siRNAs as cargo [50].

Importantly, nanoprecipitation and emulsification assisted by microfluidics are milder techniques compared to double emulsion assisted by ultrasounds (which requires sonication and extended stirring rates to allow solvent evaporation, and high temperatures could be reached), which could favor the preservation of loaded nucleic acid structure and, consequently, functionality.

For their use in gene therapy, these techniques have been mostly explored to encapsulate small nucleic acids such as siRNAs (20–25 nucleotides). However, as we mentioned before, new gene-editing systems such as CRISPR/Cas9 require vectors capable of harboring larger nucleic acids, such as high molecular weight plasmids. The physicochemical properties of these plasmids differ from small non-coding RNAs, not only in size but also in charge, structure and sensitivity to stress, being essential to validate and optimize the previously established PLGA NP synthesis protocols to encapsulate this type of nucleic acids.

In this work, we evaluated two different synthesis methods (double emulsion and nanoprecipitation) for the encapsulation of a large plasmid of 9.4 kb in PLGA NPs. Within these techniques, we tested two different batch-assisted synthesis technologies (ultrasound sonication and magnetic stirring) and compared the results with those obtained using the continuous flow microfluidics-assisted double emulsion technique. 

The efficacy of any nanotherapy largely relies on an effective internalization of nanoparticles in the target cell, which is highly dependent on their surface functionalization and particle size, being the optimal diameter between 70 and 200 nm [51,52,53]. Surface charge is also crucial for cell uptake, influencing internalization pathways and allowing endosomal escape [54,55]. Finally, in the case of nanocarriers as vehicles for gene expression vectors, DNA preservation must be achieved. Overall, all these factors make the conception of an optimal nanoparticle vehicle a complex task.

Specifically, we have compared the physicochemical properties of the nanoparticles produced (morphology, particle size, nanoparticle formation efficiency, surface charge, encapsulation efficiency and structural integrity of the loaded plasmid DNA) to show the advantages and drawbacks of the current available technologies. Notably, this manuscript will first address microfluidics-based synthesis of plasmid DNA-loaded PLGA NPs.

Taken together, our data demonstrated critical differences in biophysical properties and in vitro behavior of PLGA NPs synthesized by the previously reported protocols when loaded with high molecular weight nucleic acids. These results provide a detailed comparison of gene loading procedures for scientists in the field and highlight the need for further refinement in the use of nanocarriers as delivery vectors for high molecular weight nucleic acids, such as CRISPR/Cas gene-editing systems. 

## 2. Materials and Methods

### 2.1. Reagents

Resomer^®^ RG 503H poly(d,l-lactide-*co*-glycolide) (PLGA-COOH), dichloromethane (DCM), sodium cholate, poly(vinyl alcohol) (PVA, MW: 85,000–124,000 Da), Tween^®^ 20, Tween^®^ 80, α-Mercapto-ω-carboxy-PEG (poly(ethylene glycol)), Triton™ X-100, Pluronic^®^ F-127, dimethylformamide (DMF), 6,13-Bis(triisopropylsilylethynyl)pentacene (TIPS pentacene) and tetrahydrofuran anhydrous (THF) were purchased from Sigma-Aldrich (Darmstadt, Germany). PLGA-NH_2_ (50:50, 20 kDa) was purchased from Genochem World (Valencia, Spain).

Plasmid DNA (pDNA) was obtained from DH5*α* bacteria (Thermo Fisher Scientific, Waltham, MA, USA) transformed with DC-RFP-SH01 plasmid (pRFP plasmid) (GeneCopoeia™, Rockville, MD, USA). pRFP is 9431 bp long.

Motor neuron-like NSC-34 cell line, used for the cellular internalization assay of pDNA-loaded PLGA NPs, was purchased from Cedarlane Laboratories and grown in high glucose (4.5 g/L) Dulbecco’s modified Eagle’s medium (DMEM), 10% (*v*/*v*) fetal bovine serum (FBS), penicillin (100 U/mL) and streptomycin (100 μg/mL). These cells grow in adherence when seeded on a plastic surface coated with collagen A and incubated at 37 °C, 95% relative humidity and 5% CO_2_.

### 2.2. Isolation of Plasmid DNA

pDNA was isolated from bacterial cell culture grown O/N in Luria–Bertani (LB) medium and extracted using the Endofree^®^ Plasmid Mega Kit (QIAGEN, Germantown, MD, USA) according to the manufacturer’s protocol.

### 2.3. Effects of Surfactants on pDNA Conformation Assay

The pDNA was incubated for 3 h at RT with the following solvent mixtures: DCM, DCM + 0.01% sodium cholate, DCM + 0.3% sodium cholate, DCM + 0.6% sodium cholate, DCM + 1% sodium cholate, DCM + 1% PVA, DCM + 1% (41% Tween 80 + 59% Tween 20) and DCM + 1% Triton X-100, mimicking the conditions during the double emulsion procedure. Subsequent loading onto a 1% agarose gel in TBE buffer (8.9 mM Tris, 8.9 mM boric acid, 0.2 mM EDTA, pH 8.4) containing Gel Green nucleic acid stain (Biotium, Fremont, CA, USA) and electrophoresis resolved the different conformations present in each sample. Purified pDNA stock in DNAse-free Milli-Q water was used as the control, confirming the exclusive presence of the supercoiled functional conformation in the sample. Adequate separation of the bands for supercoiled, linear, and open circular plasmid DNA was obtained by running the electrophoresis gel for at least 40 min at 60 V. Images were acquired using a UV transilluminator (Vilber Lourmat, TCX-20C, Collégien, France).

### 2.4. Synthesis of DNA-Loaded PLGA Nanoparticles by the Double Emulsion Solvent Evaporation Method Assisted with a Batch-Ultrasound Procedure (Ultrasound-Assisted w/o/w)

A total of 50 mg of PLGA-COOH was dissolved in 3 mL of DCM using an ultrasonic bath. Subsequently, 1 mL of aqueous phase (900 μL of 33.33 μg/mL plasmid in Milli-Q water plus 100 μL of 0.1% sodium cholate (*w*/*v*)) was added at 4 °C and sonicated in an ice bath with a fine-probe sonicator (Branson Digital Sonifier^®^, Fisher Scientific, Madrid, Spain) for 20 s and at 40% amplitude. An amount of 8 mL of 1% (*w*/*v*) sodium cholate was added and the mixture was sonicated again for 40 s and at 40% amplitude. After addition of 12 mL of 0.3% (*w*/*v*) sodium cholate, the nanoparticle solution was allowed to evaporate in a fume hood for 3 h at RT. Finally, DNA-loaded PLGA nanoparticles were centrifuged at 8500× *g* rpm for 15 min at RT, supernatant was decanted and saved for quantification of DNA encapsulation and the pellet was resuspended in 2 mL of Milli-Q water. Hereon, this method will be named “standard ultrasound-assisted double emulsion”.

A modified double emulsion method, hereon named the “modified ultrasound-assisted double emulsion” method, was included in this study. This variant used the above-explained synthesis methodology with the following modifications. pDNA was previously incubated with PEG (ratio 1:15 *w:w*), which has been previously reported to improve plasmid structure preservation during the synthesis process [56], for 1 h at room temperature (RT). In addition, sonication times were reduced to 10 s and 20 s to minimize pDNA exposure time to sonication while still allowing NPs to form. 

### 2.5. Synthesis of DNA-Loaded PLGA Nanoparticles by the Double Emulsion Solvent Evaporation Method Assisted with Microfluidics (Microfluidics-Assisted w/o/w)

The microfluidic system used was similar to the one previously described in our group (Larrea et al., 2017) to produce *w*/*o*/*w* double emulsions in continuous flow. Two slit interdigital micromixers were assembled serially to promote the sequential formation of the double emulsion. In our previous manuscript, we loaded a gold precursor and sodium citrate in the first micromixer (w/o emulsion) and after forming the double *w*/*o*/*w* emulsion in the second micromixer, the emulsion was heated to promote the reduction of Au ionic precursor into Au nanoparticles. This process yielded 192 ± 58 nm-diameter PLGA NPs, whose lumens were selectively loaded with Au NPs. The slit interdigital micromixer with an inner volume of 8 µL (Micro4Industries GmbH, Mainz, Germany) was interfaced with one Polytetrafluoroethylene (PTFE) tube (OD = 1/1600, ID = 1 mm). Inlet streams in the microfluidic system were labeled as I1, I2 and I3, I1 and I2 being mixed in the first micromixer, and the resulting stream being mixed with I3 in the second micromixer. Reagents were injected using three syringe pumps (Harvard Apparatus, Holliston, MA, USA). 

**Figure 1:**
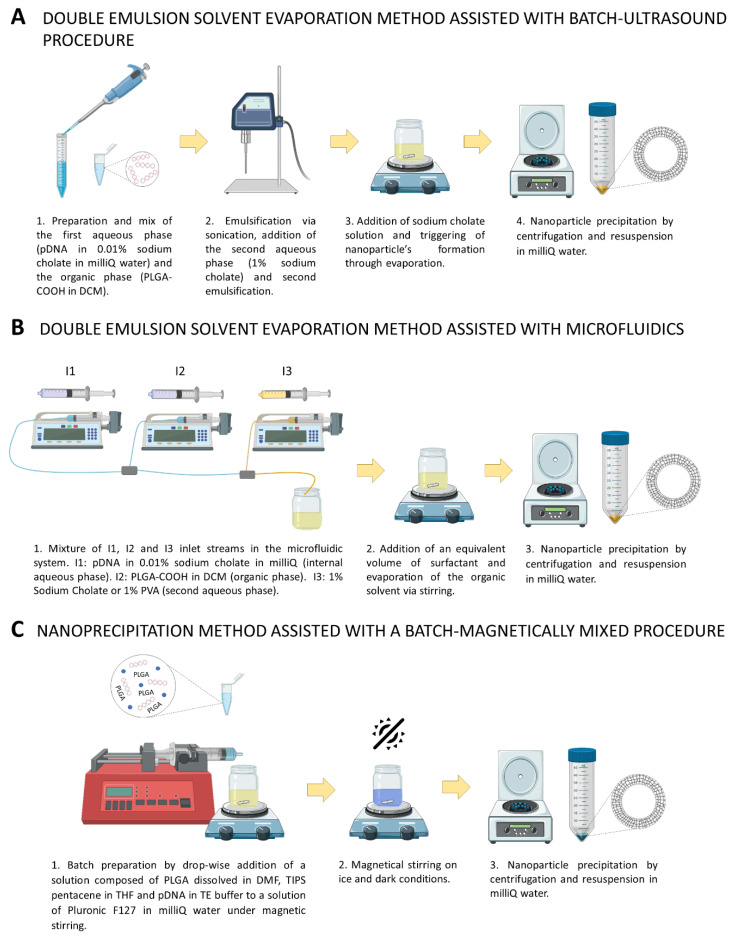
**Graphical abstract for the different synthesis methods tested**. (**A**) Protocols followed for nanoparticle synthesis and pDNA encapsulation using double emulsion method assisted with batch ultrasound and (**B**) microfluidics procedures (**C**) Protocol followed in nanoprecipitation method assisted with a batch magnetically mixed procedure.

Synthesis conditions were adapted from previous research by selecting a different organic solvent and cargo (gold nanoparticle precursors were substituted by pDNA) as follows. The I1 stream is the internal aqueous phase and consists of 0.01% (*w*/*v*) sodium cholate and 30 μg of pDNA dissolved in 3 mL of Milli-Q water. The organic stream I2 was prepared by adding 50 mg of PLGA-COOH to 20 mL of DCM. I1 and I2 were injected in the first micromixer with a flow rate of 4.5 and 13.5 mL/min, respectively. The resulting primary emulsion (*w*/*o*) produced after mixing I1 and I2 was injected in the second micromixer to form the double emulsion (*w*/*o*/*w*) with the aid of the I3 stream. At this point, we introduced the surfactant used as a synthesis variable. Thus, two variants were established for this method: one using sodium cholate and the other using PVA. The choice of these two surfactants was made based on the results of the effects of surfactants on pDNA conformation assay. Hence, the I3 stream was prepared by dissolving 1% sodium cholate (microfluidics-assisted *w*/*o*/*w* with sodium cholate) or 1% PVA (*w*/*v*) (microfluidics-assisted *w*/*o*/*w* with PVA) in 50 mL of Milli-Q water, and injected at a flow rate of 36 mL/min. The same volume of 0.3% sodium cholate (for microfluidics-assisted *w*/*o*/*w* with sodium cholate) or 1% PVA (for microfluidics-assisted *w*/*o*/*w* with PVA) (*w*/*v*) was added to the final collected emulsion. Finally, the organic solvent was evaporated under continuous stirring at 600 rpm in an open flask for 3 h, DNA-loaded PLGA nanoparticles were centrifuged at 8500× *g* rpm for 15 min at RT, supernatant was collected for quantification of pDNA encapsulation, and the pellet was resuspended in Milli-Q water (Figure 1).

### 2.6. Synthesis of DNA-Loaded PLGA Nanoparticles by Nanoprecipitation Method Assisted with a Batch-Magnetically Mixed Procedure

The DNA-loaded PLGA nanoparticles were prepared by the modified nanoprecipitation method previously described by Jo et al. (2020) with a slight modification in the nanoparticle precipitation step. Briefly, 100 mg Pluronic F127 were dissolved in 20 mL Milli-Q water by vortexing, followed by 30 min of sonication in an ultrasonic bath and 30 min of magnetic stirring at 600 rpm. During magnetic stirring, solutions of PLGA dissolved in DMF (44.48 mg/mL) and TIPS pentacene dissolved in THF (0.667 mg/mL) were prepared separately. Subsequently, 562 μL of PLGA stock, 562 μL of TIPS pentacene stock and 126 μL of plasmid DNA solution in TE buffer (4.07 mg/mL) were combined in a 1.5 mL centrifuge tube and gently mixed until visually homogenous. The resulting mixture was added drop-wise (30 mL/h) with an infusion pump to the aqueous Pluronic F-127 solution while magnetically stirring at 600 rpm. The final solution was left, stirred for 5 h on ice, covered to protect TIPS pentacene from light exposure, and finally centrifuged at 4 °C and 8500× *g* for 15 min (Figure 1). Supernatant was collected for quantification of pDNA encapsulation and the pellet was resuspended in 2 mL of Milli-Q water and stored at 4 °C. 

For this experiment, two types of PLGA were tested: cationic amino-terminated PLGA (PLGA-NH_2_) and anionic carboxyl-terminated PLGA (PLGA-COOH).

### 2.7. Nanoparticle Morphology

Nanoparticle morphology was observed by a CSEM-FEG Inspect 50 Field Emission Scanning Electron Microscope (SEM) (FEI Company, Hillsboro, OR, USA) at high vacuum and with an acceleration voltage of 20kV. For sample preparation, a drop of the corresponding resulting nanoparticle dispersion was placed on a slide, fixed with carbon tape to an SEM microscope holder, air-dried and sputtered with Palladium to promote electron conduction. 

### 2.8. Particle Size Measurement

In order to define the resulting particle size, particles were measured using transmission electron microscopy (TEM) (Tecnai F30, FEI Company, Hillsboro, OR, USA). Organic staining of the nanoparticle dispersion was carried out using phosphotungstic acid dissolved in Milli-Q water (30 mg/mL) mixed at a 1:1 (*v*:*v*) ratio with the polymer sample and incubated for 45 min. After washing to remove the phosphotungstic acid excess, samples were placed on TEM grids (carbon fiber copper microscopy stand, Electron Microscopy Sciencies FCF200-Cu) and images were taken by a FEI Tecnai T20 microscope. Images were captured at different magnifications and processed using ImageJ software v.1 (publicly available at https://imagej.nih.gov/ij/ (accessed on 7 October 2021)) to determine the Minimum Feret Diameter of each particle. At least 100 nanoparticles were measured from each sample. Results were represented as diameter size distributions and mean diameter ± SD. 

### 2.9. Nanoparticle Synthesis Yield

To quantify the PLGA NP synthesis yield, 3 metal pans were weighed in triplicate to minimize the error (TYPUYA ultramicrobalance, TYP MEDICIONES™, Albacete, Spain). Subsequently, 25 μL of the corresponding NPs dispersion were added into each well. The solution was allowed to evaporate overnight in an oven at 80 °C before metal pans were reweighed to calculate PLGA NP concentration by mass balance.

Total yield of PLGA NPs was calculated from PLGA NP concentration and related to the initial amount of PLGA polymer used for the synthesis by applying the following equation:

$$\mathrm{NP}\;\mathrm{yield}\;\left(\%\right)=\frac{\;\mathrm{PLGA}\;\mathrm{NP}\;\mathrm{concentration}\;\times \;\mathrm{total}\;\mathrm{volume}\;\mathrm{of}\;\mathrm{PLGA}\;\mathrm{NPs}}{\mathrm{initial}\;\mathrm{amount}\;\mathrm{of}\;\mathrm{PLGA}\;\mathrm{polymer}}\times 100$$

### 2.10. Nanoparticle Zeta Potential

Zeta potential was determined by Phase Analysis Light Scattering using a Brookhaven 90Plus Particle Size Analyzer and the BIC PALS Zeta Potential Analyzer Software (both available from Brookhaven Instruments Corp, NY, USA). Parameter settings were as follows: number of runs (5), number of cycles (30), resuspension liquid (water). pH and particle nature were also set. For readings to be valid, conductance and sample count rate were higher than 20 μS and 320 kcps, respectively.

### 2.11. Analysis of pDNA Encapsulation Efficiency and Structural Integrity 

pDNA encapsulation efficiency was calculated from the following equation:

$$\mathrm{pDNA}\;\mathrm{encapsulation}\;\mathrm{efficiency}\;\left(\%\right)=\frac{\mathrm{amount}\;\mathrm{of}\;\mathrm{pDNA}\;\mathrm{loaded}\;\mathrm{into}\;\mathrm{PLGA}\;\mathrm{NPs}\;}{\mathrm{total}\;\mathrm{pDNA}\;\mathrm{amount}\;\mathrm{added}}\times 100$$

The amount of DNA loaded into the PLGA nanoparticles was determined by measuring the difference between the total amount of pDNA added during synthesis and the amount of free pDNA recovered from the supernatant after nanoparticle centrifugation. The concentration of pDNA in the supernatant was quantified using the Qubit™ 1X dsDNA High Sensitivity Kit (Thermo Fisher Scientific Q33231, Waltham, MA, USA) according to the manufacturer’s instructions and a Qubit™ 4 Fluorometer device (Thermo Fisher Scientific, Waltham, MA, USA). 

The structural integrity of the loaded plasmid was evaluated by agarose gel electrophoresis analysis. For this purpose, 20 μL of PLGA NPs was centrifuged at 20,000× *g* for 45 min and NP pellets were incubated in water for 24 h at 4 °C after synthesis, facilitating PLGA hydrolysis and release of the encapsulated plasmid into the aqueous phase. Aqueous phases containing the released pDNA were loaded into a 1% agarose gel. Conditions for gel electrophoresis and image acquisition were as described above.

As shown in Supplementary Figure S1, plasmids can adopt various spatial conformations leading to supercoiled, linear, relaxed, nicked open-circular and denatured supercoiled isoforms. The supercoiled DNA index (SCI) was defined as the integrated density of the supercoiled DNA band over the total integrated density of all bands present in the sample. Similarly, the relative presence of each plasmid isoform can be calculated by dividing the integrated density of the intended band by the total density of all bands present in the sample.

The integrated density of each of the SCI bands is calculated by subtracting the net background integrated density for the same area from the net integrated intensity of the given band. 

### 2.12. DNA Release Profile 

Before quantifying the concentration of pDNA released with the Qubit™ 1X dsDNA High Sensitivity Kit, PLGA NPs were pelleted by centrifugation at 20,000× *g* and RT for 45 min and supernatant was collected for DNA quantification. The pDNA release rate is expressed as a percentage of the amount of encapsulated plasmid using the following equation:

Release rate %= concentration of released pDNAamount of pDNA loaded into PLGA NPstotal volume of PLGA NP dispersion × 100

### 2.13. Cell Viability Assay

NSC-34 cells were seeded at 5000 cells/well in a 96-well plate and incubated with pDNA-loaded or empty nanoparticles synthesized by nanoprecipitation with PLGA-NH_2_ at different concentrations (0.01 mg/mL, 0.05 mg/mL, 0.1 mg/mL, 0.2 mg/mL and 0.4 mg/mL) for 24 h at 37 °C and 5% CO_2_. Consequently, resazurin (Sigma, R7017) was added to each well at a final concentration of 0.1 mM and incubated for 6 h. Finally, fluorescence was measured in a Synergy 4 spectrofluorimeter (BioTec Instruments, Winooski, VT, USA) using a 530 nm excitation filter and 590 nm emission filter.

Controls included in this assay were untreated cells (negative control), cells incubated for 12 h in phosphate buffered saline (PBS) (induced cell death, positive control) and PLGA NPs diluted in growth medium at 0.01 mg/mL, 0.05 mg/mL, 0.1 mg/mL, 0.2 mg/mL and 0.4 mg/mL in wells with no cells (background). 

### 2.14. Cellular Internalization Assay

#### 2.14.1. Quantification of pDNA-Loaded PLGA NPs Cell Internalization by Fluorescence

NSC-34 cells were seeded at 280,500 cells/well in 6-well plates and cultured overnight at 37 °C. The next day, 0.2 mg/mL of pDNA-loaded NPs synthesized by nanoprecipitation with amino-terminated PLGA were added to the medium and incubated with cells for 24 or 48 h (three replicates per condition). Non-treated cells were used as controls. After incubation, cells were washed twice with PBS to remove non-internalized NPs. Subsequently, cells were mechanically removed with a scraper, counted and resuspended in 500 µL of distilled water for lysis. This lysis volume, corresponding to each p6 well was distributed into a 96-well plate (5 replicates from each lysate). In parallel, a calibration curve was prepared from TIPS-loaded PLGA NPs at different concentrations in distilled water. Finally, the fluorescence of the plate was recorded in a Synergy4 spectrofluorometer and the amount of internalized PLGA NPs in each cell was calculated by interpolation into the standard curve. 

#### 2.14.2. Visualization of pDNA-Loaded PLGA NPs Cell Internalization by Confocal Microscopy

NSC-34 cells were seeded at 15,000 cells/well in a µ-slide 8-well ibiTreat plate and cultured overnight at 37 °C. Consequently, cells were treated with 0.2 mg/mL of pDNA-loaded NPs synthesized by nanoprecipitation with amino-terminated PLGA and fixed with 4% paraformaldehyde in PBS at different time points (4 h, 8 h, 12 h, 24 h and 48 h). Non-treated cells were used as control.

After fixation, cells were permeabilized and stained with a solution of 1% BSA (*w*/*v*), 0.1% saponin (*w*/*v*) and 1:200 of Alexa Fluor™ 488 phalloidin in PBS for 1 h in dark conditions, washed with PBS and prepared in confocal microscopy, adding mounting medium with DAPI (10 µL of DAPI in 16 drops of Vectashield^®^ Antifade Mounting Medium, Vector Laboratories). Finally, pDNA-loaded PLGA NPs cell uptake was analyzed using a confocal microscope (Spectral Zeiss LSM 880, Carl Zeiss Microscopy GmbH, Jena, Germany, available at the Microscopy and Imaging service of the Biomedical Research Center of Aragón).

### 2.15. Cell Transfection Assays

NSC-34 cells were seeded in 24-well plates (100,000 cells/well), pDNA-loaded nanoparticles corresponding to 2 μg of encapsulated plasmid were added into the culture medium and incubated for 72 h (24 h uptake + 48 h expression). Cells transfected with lipofectamine and 2 μg of pDNA following manufacturer’s protocol and allowing 48 h for plasmid expression were used as a positive control. A negative control with untreated cells was also included. Each condition was performed in duplicate.

For immunofluorescence assay, cells were fixed with 10% formalin for 30 min, washed with 1X PBS twice and 500 μL of 1X PBS and 0.5 μL of DAPI were added to each well. Cells were checked for transfection with an inverted fluorescence microscope (IX81 Olympus Inverted Optics Microscope, Olympus Europa Holding GmbH, Hamburg, Germany, available at the Microscopy and Imaging service of the Biomedical Research Center of Aragón).

### 2.16. Statistical Analysis

Results are shown as the mean value ± the standard deviation. To establish significant differences between the three pDNA-loaded PLGA NP synthesis techniques, a three-way ANOVA statistical analysis was performed for each of the numerical properties analyzed in the present work. Significant differences were considered with a 95% interval of confidence (*p* < 0.05) or 99% (*p* < 0.01), as indicated in each case.

## 3. Results and Discussion

As mentioned above, many factors can affect the efficacy of nanocarriers as vehicles for gene expression. Among these factors, nanoparticle size, surface charge, loading capacity, cargo integrity preservation and release are of particular relevance, which will be analyzed throughout this work for each of the synthesis procedures.

### 3.1. Effects of Different Solvents on pDNA Conformation

Although the three PLGA NP synthesis procedures studied (ultrasound- and microfluidics-assisted *w*/*o*/*w* and nanoprecipitation) rely on different physical principles, they all share the use of organic solutions containing dissolved PLGA, aqueous buffers with hydrophilic cargo (i.e., pDNA) and surfactants to promote emulsion formation, stability, and size control. DCM has been widely used as a PLGA solvent; however, the nature and concentration of aqueous-based buffers is highly variable among protocols [42]. Solvent type used is critical for structural integrity of the plasmid, potentially modifying the natural supercoiled isoform of pDNA into a linear, relaxed or denatured conformation. Supercoiled conformation possesses the highest transfection capacity and is considered to be transcriptionally active, while other isoforms result in a significant loss of functionality.

Given the high molecular weight of our plasmid (9.4 kb) and the importance of pDNA structural integrity to maintain functionality, we first aimed at analyzing the effects of the most commonly used organic and aqueous buffers on plasmid conformation.

**Figure 2:**
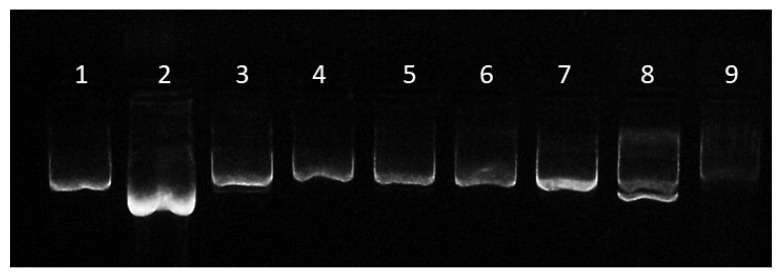
**Effect of synthesis solvents on pDNA structure stability**. Image shows the migration of pRFP plasmid in (**1**) Milli-Q water, (**2**) DCM, (**3**) DCM + 0.01% sodium cholate, (**4**) DCM + 0.3% sodium cholate, (**5**) DCM + 0.6% sodium cholate, (**6**) DCM + 1% sodium cholate, (**7**) DCM + 1% PVA, (**8**) DCM + 1% (41% Tween 80 + 59% Tween 20) and (**9**) DCM + 1% Triton X-100 after 3 h of incubation at RT.

Results showed how incubation with DCM alone produces a conformational change into denatured supercoiled pDNA (Figure 2, lane 2), leading to a faster migration in the agarose gel compared to the untreated control. This is probably due to the low polarity of DCM, so that pDNA, which is a hydrophilic and polar molecule, acquires a conformation that minimizes exposure to this organic solvent. This effect is absent when pDNA is exposed to a mixture that, aside from DCM, contains an aqueous solution such as sodium cholate or PVA (Figure 2, lanes 3 to 6 and 7, respectively). Conversely, incubation with DCM plus 1% (41% Tween 80 and 59% Tween 20) induced the denatured supercoiled conformation in 50.18% of the plasmid (Figure 2, lane 8). Likewise, incubation with Triton X-100 also produced a conformation change of the pDNA, in this case to a relaxed form (Figure 2, lane 9). Interestingly, our results suggest that most non-ionic surfactants (Tween 80, Tween 20 and Triton X-100, but not PVA) fail to preserve the plasmid supercoiled conformation on exposure to DCM.

Our findings differ from those described by Doolaanea and colleagues, who reported Triton X-100 as a good pDNA structure preserver [42]. These discrepancies might be due to distinct plasmid sizes or our direct exposure of pDNA to the solvent mixture vs. Doolaanea’s plasmid structure analysis in the context of a synthesis reaction.

Given our results, PVA and sodium cholate were selected as the best plasmid structure preservers in our synthesis conditions and were further used for PLGA NP synthesis by ultrasound- and microfluidics-assisted *w*/*o*/*w* procedures.

### 3.2. Morphology, Particle Size, Concentration and Zeta Potential of DNA-Loaded PLGA Nanoparticles

**Figure 3:**
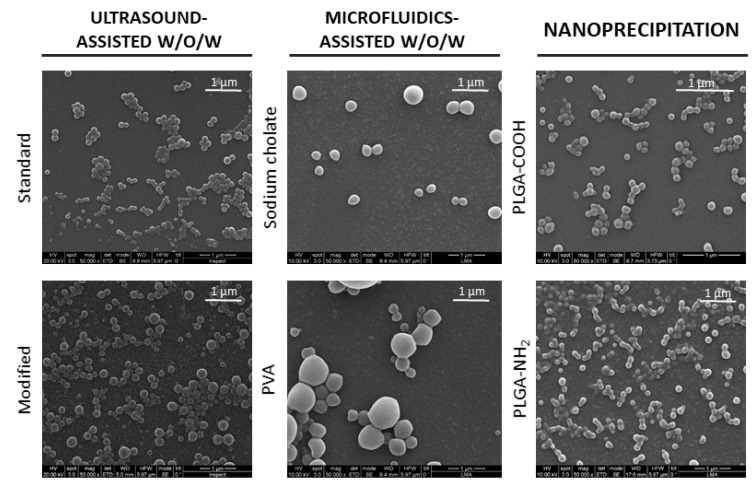
**Morphology of pDNA-loaded nanoparticles**. Scanning electron microscopy images of nanoparticles produced by the different synthesis procedures evaluated in this work.

SEM imaging confirmed that all three synthesis procedures resulted in spherical and intact nanoparticle formation (Figure 3). 

As mentioned before, the efficacy of any nanotherapy relies on an effective internalization of nanoparticles in the target cell, which in turn depends greatly on their size and surface charge (zeta potential). It has been proven that PLGA NPs with a size between 70 and 200 nm are stable in circulation, unlike those with larger or smaller sizes [51,52]. Nanoparticles smaller than 70 nm have been reported to accumulate in the liver, where they are rapidly metabolized. On the other hand, those larger than 200 nm are preferentially located in the spleen, where they are phagocytosed by macrophages [53]. 

**Figure 4:**
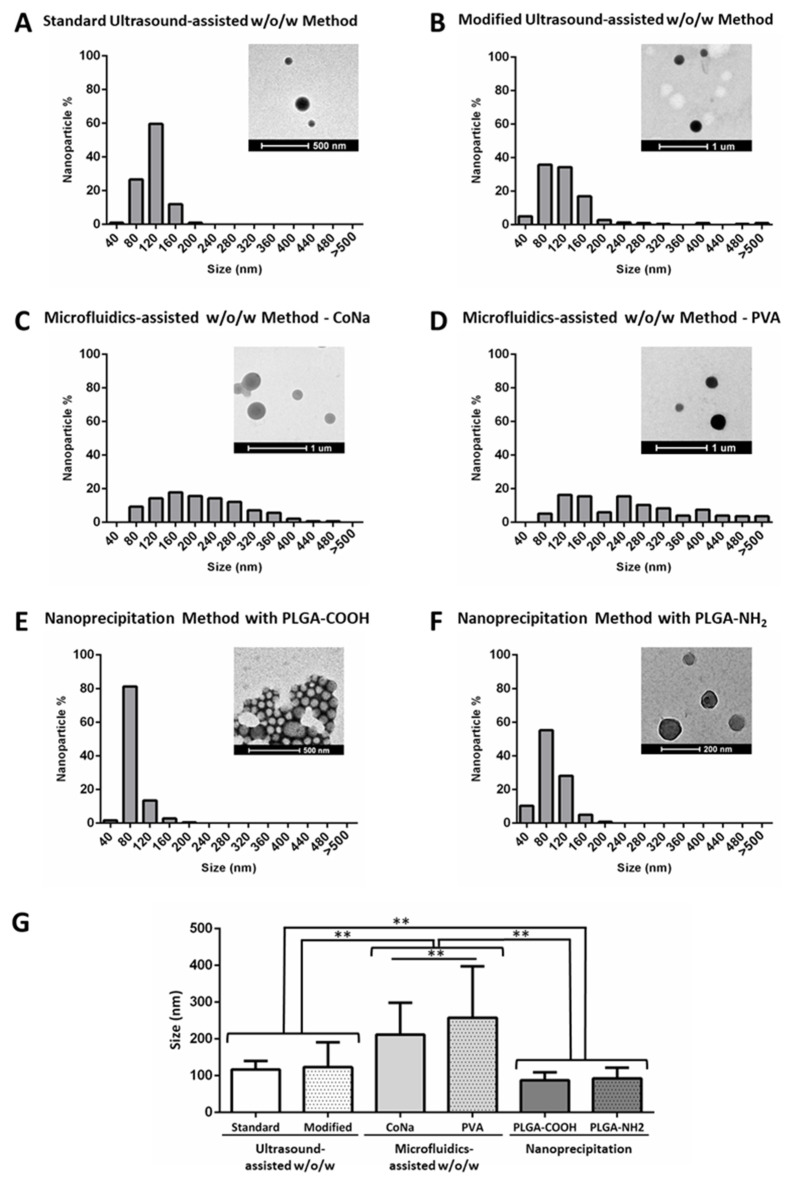
**Relative size distribution of pDNA-loaded nanoparticles produced by the different synthesis methods evaluated in this work**. PLGA nanoparticles were visualized by transmission electron microscopy (TEM) and size distribution of PLGA NPs was represented in percentages. Data were obtained from the measurements of diameters in TEM images of at least 100 dry nanoparticles for each synthesis method with ImageJ software. (**A**) Standard ultrasound-assisted double emulsion method, (**B**) modified ultrasound-assisted double emulsion method, (**C**) microfluidics-assisted double emulsion method with sodium cholate as surfactant, (**D**) microfluidics-assisted double emulsion method using PVA as surfactant, (**E**) nanoprecipitation method with carboxyl-terminated PLGA, (**F**) nanoprecipitation method with amino-terminated PLGA, (**G**) diameter comparisons among the different synthesis methods. * *p* < 0.05, ** *p* < 0.01.

As measured from TEM images, in this order, our results showed that microfluidic technology resulted in larger nanoparticles (212 ± 87 and 258 ± 140 nm for sodium cholate and PVA syntheses, respectively) as compared to ultrasound-assisted *w*/*o*/*w* and nanoprecipitation techniques, the latter two being within the desired 70–200 nm size range for this study. Ultrasound-assisted *w*/*o*/*w* resulted in an intermediate particle size (116 ± 24 and 123 ± 67 for standard and modified procedures, respectively), nanoprecipitation being the best method to obtain smaller nanoparticles (88 ± 21 and 93 ± 29 nm for PLGA-COOH and PLGA-NH_2_, respectively) (Table 1, Figure 4). No statistically significant differences were found among nanoparticles synthesized by any of the variants of ultrasound-assisted double emulsion (standard vs. modified) and nanoprecipitation (PLGA-COOH vs. PLGA-NH_2_) methodologies. However, significant differences were found between NPs when synthesized by microfluidics-assisted double emulsion with sodium cholate or PVA (Table 1, Figure 4).

NP size polydispersity for both double emulsion and nanoprecipitation methods was lower compared to that obtained by microfluidics (Table 1, Figure 3 and Figure 4). Interestingly, polydispersity of NPs synthesized through emulsion assisted by microfluidics was higher than in our previous studies [43], even using the same fluid dynamic parameters. This might be explained by the use of DCM as a PLGA solvent in the organic phase instead of ethyl acetate, as the latter has been previously reported to cause DNA degradation. Different densities for both DCM (1.33 g/cm^3^) and ethyl acetate (0.90 g/cm^3^), different viscosities (0.413 cP and 0.428 at 25 °C for DCM and ethyl acetate, respectively) and different polarity (3.1 and 4.4 polarity index for DCM and ethyl acetate, respectively) likely affected shear stress, channel wettability, mixing efficiency and, thus, emulsion control over the synthesis process, contributing to discrepancies in the resulting polydispersity between both works. These results also evidence the need for specific NP synthesis protocols and reagents for large pDNA encapsulation, both to preserve pDNA structure and control NP morphology. 

In terms of NP formation efficiency, double emulsion-based (*w*/*o*/*w*) methods have a NP yield of approximately 40% referred to PLGA input. This yield is lower (27.20 ± 10.68%) in the case of microfluidics-assisted *w*/*o*/*w* synthesis method using sodium cholate as the surfactant (Table 1). Moreover, nanoprecipitation procedure with amino-terminated PLGA also showed relatively low efficiency (28.48 ± 10.57%), whereas values up to 54.96% were reached when carboxyl-terminated PLGA was used.

On the other hand, NP surface charge, also known as zeta potential, influences the stability of the particles in suspension and, thus, in the bloodstream. Zeta potentials close to zero (isoelectric point) promote aggregation, while absolute values higher than 30 mV make NPs stable in suspension. Moreover, the sign of the charge and its value also determine the way in which the particles will interact with the plasmatic membrane of cells and, therefore, will influence their internalization [57]. Although better cell internalization has been observed when the nanoparticles are positively charged (due to their interaction with the negative charge residues of the cytoplasmic membrane), negatively charged nanoparticles can also be internalized. Current hypotheses suggest that this internalization occurs through receptor-mediated endocytosis mechanisms [58] or interaction with positively charged specific sites on the cell membrane [59]. In addition to internalization, negatively charged PLGA NPs have been shown to undergo rapid endo-lysosomal escape due to the selective inversion of their surface charge (from anionic to cationic) within the endo-lysosomal compartment [54]. This allows the NPs to interact with the endo-lysosomal membrane and escape into the cytosol, avoiding intra-lysosomal cargo degradation and allowing sustained intracellular release of a wide variety of therapeutic agents, including macromolecules such as DNA.

In this work, all synthesis methodologies yielded negatively charged PLGA nanoparticles with zeta potentials ranging from −33 to −57 mV, being more negative in the modified ultrasound-assisted double emulsion method (−57.32 ± 10.16 mV) and the nanoprecipitation using amino-terminated cationic PLGA method (−46.22 ± 12.49 mV) (Table 1). These differences were statistically significant for the modified ultrasound-assisted *w*/*o*/*w* (*p* < 0.01 for all comparisons except for nanoprecipitation method using cationic PLGA, *p* = 0.1653), while they remained only a trend for the case of nanoprecipitation with PLGA-NH_2_.

Given the cationic nature of amino-terminated PLGA, zeta potential would be expected to be positive; however, electrostatic interactions between the high molecular weight pDNA and PLGA-NH_2_ likely favor accumulation of the plasmid on the surface of the nanoparticles, being responsible for the negative charge observed for the pDNA-loaded PLGA NPs. DNA is a negatively charged molecule, this charge being normally related to size (the longer, the more negative). Accordingly, it is reasonable to assume that the larger the amount of plasmid trapped in the particle, the greater the amount of pDNA would remain on NPs surface and, therefore, the more negative its surface charge would tend to be. This is in agreement with the tendency observed for a negative correlation between the Z-potential and the encapsulation yield obtained for each of the replicates of the different syntheses (Supplementary Figure S2). This correlation was also observed when pooling data from the different synthesis methods and polymers, although the greater variability introduced prevented statistical significance.

### 3.3. pDNA Encapsulation Efficiency and Structural Integrity Analysis of the Loaded DNA in PLGA Nanoparticles

PLGA nanoparticles have demonstrated to have loading capacity for many different biomolecules, including nucleic acids, proteins and diagnostic agents in addition to the small molecules that have traditionally been used as therapeutic drugs [11]. 

Encapsulation efficiency of drugs described in the literature is highly variable, ranging from 10 to 96% depending on the nanoparticle type and its physicochemical characteristics, the synthesis process and the drug nature [37,41,60,61]. 

Our study also reflects this variability, obtaining a range of values between 20 and 60% of encapsulation efficiency for plasmid DNA (Table 1), being significantly higher for NPs synthesized by microfluidics-assisted *w*/*o*/*w* with PVA (50.91 ± 23.27%) (*p* < 0.01) and nanoprecipitation using PLGA-NH_2_ (56.84 ± 16.09%) (*p* < 0.01). Interestingly, and as opposed to double emulsion procedures, nanoprecipitation synthesis with carboxyl-terminated PLGA resulted in no encapsulation. This might be explained by electrostatic repulsive forces between the polymer and the pDNA during nanoprecipitation, unlike double emulsion and microfluidics, where other forces such as sonication-based emulsification and interfacial tension within microreactor channels are in play to promote encapsulation. 

**Figure 5:**
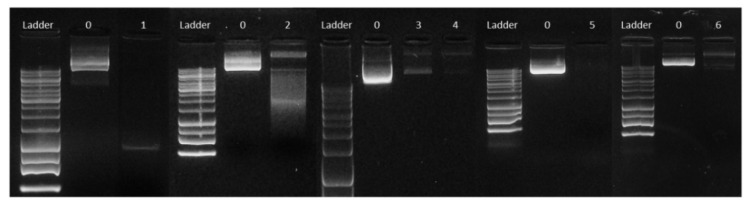
**Effect of the different synthesis procedures on pDNA structure**. Standard (**1**) and modified (**2**) ultrasound-assisted double emulsion procedures result in total and partial plasmid degradation, respectively. Microfluidics-based double emulsion synthesis does not degrade the pDNA but does alter its structure so that it adopts a linear and relaxed isoform. This occurs both in the case of using sodium cholate (**3**) and PVA (**4**) as surfactants. Finally, the nanoprecipitation method with carboxyl-terminated PLGA does not lead to pDNA encapsulation (**5**), whereas with amino-terminated PLGA, the plasmid preserves 41.97% of its functional structure, leaving the remaining 59.03% in a relaxed conformation (**6**). Samples were run next to the stock used for the corresponding synthesis as control (**0**).

Preservation of structural integrity and functionality of the encapsulated genetic material is critical when using nanoparticles as gene therapy vectors. In this sense our results showed that the ultrasound-assisted double emulsion method resulted in pDNA cleavage into small fragments as a consequence of the fine-probe sonicator action (Figure 5, lane 1 and Supplementary Figure S3). Modification of sonication times and amplitude did not prevent plasmid degradation (data not shown). In this sense, incubation with PEG has been claimed to protect pDNA integrity during batch ultrasound-assisted double emulsion synthesis [56]. However, in these conditions our data showed an extensive plasmid degradation (73.64% of total pDNA) and unfolding into a nicked open-circular isoform (18.86% of total pDNA) (Figure 5, lane 2). 

Alternatively, some authors have included the use of homogenizers instead of fine probe sonication for emulsification, reporting open circular and supercoiled conformations [42]. However, in our hands, the use of homogenizers did not preserve the structure of encapsulated high molecular weight pDNA (Supplementary Figure S3, lanes 2, 3 and 4) and resulted in larger nanoparticles (data not shown).

Despite the fact that the ultrasound-assisted double emulsion solvent evaporation method has been reported for the synthesis of functional nucleic acid-loaded PLGA nanoparticles, nucleic acid structure is not commonly analyzed, and sonicators or homogenizers used to prepare the emulsions can induce mechanical shear. This type of stress leads to nucleotide sequence disruption or changes the supercoiled functional conformation of the plasmid into open circular or linear isoforms [62,63]. 

Moreover, these studies are usually carried out using medium to low molecular weight plasmids (typically around 5.5 kb but up to 7.3 kb), [35,42,64,65,66], which might be less sensitive to unfolding or sonication tearing than larger nucleic acid chains. In fact, to our knowledge, this is the first study validating ultrasound- or microfluidics-assisted double emulsion encapsulation of large pDNAs (9.4 kb), similar to those encoding the CRISPR/Cas gene editing system (which are typically about 9.10 kb in size) [2]. 

On the other hand, microfluidics-based methods do not rely on sonication for emulsification, but on the flow through small-sized microchannels. Encapsulation of pDNA using microfluidics-assisted double emulsion resulted in high shear stress that modified the supercoiled structure of the plasmid.

Specifically, microfluidics-based synthesis using sodium cholate exhibited 64.80 ± 1.99% and 35.20 ± 1.99% for linear and nicked open-circular isoforms (Table 1 and Figure 5, lane 3), while using PVA resulted in a 45.01 ± 26.28% and 54.99 ± 26.28%, respectively (Table 1 and Figure 5, lane 4). Both isoforms are reported to halt cell transfection [67], which, together with the large plasmid size, which also reduces “per se” transfection efficiency [3], contributed to the lack of pDNA expression in vitro cell experiments (data not shown). 

Given that reagents used for double emulsion were common between batch ultrasound and microfluidics-assisted syntheses and did not affect plasmid structure (Supplementary Figure S3), this is likely a consequence of mechanical stress when passing through the microchannels. Unfortunately, reducing shear stress by decreasing fluid flow rates has been observed to increase nanoparticle size and polydispersity [68].

In turn, nanoprecipitation with amino-terminated PLGA preserved part of the supercoiled and functional isoform of the plasmid (approximately 42%), while the remaining 58% showed a relaxed conformation (Figure 5, lane 6). Relaxed conformation is likely caused by exposure to the combination of synthesis reagents (Supplementary Figure S4). However, given the high encapsulation efficiency obtained (56.84 ± 16.09%, which is equivalent to 4.12 ± 1.09 wt.%) and, as long as NPs are non-cytotoxic and efficient cell internalization and cargo release are achieved, these nanoconstructs have the potential to induce pDNA cell expression in vitro. Hence, NP cytotoxicity, cell internalization and cargo release were further analyzed.

### 3.4. DNA Release Profile, In Vitro Citotoxicty and Cellular Internalization of pDNA-Loaded NPs

Tracking of pDNA release from the nanoparticles synthesized by nanoprecipitation with amino-terminated PLGA showed that during the first 24 h at 4 °C and 37 °C, only about 2.2% and 4% of the encapsulated pDNA was released, respectively. This result might be explained by the electrostatic interactions between the large pDNA and positively charged amino-terminated PLGA in an aqueous environment, which could be strong enough to prevent plasmid release from the particle or fast re-adsorption after release as previously reported for other polymers and cargoes [69,70,71]. 

Preliminary studies from our group suggest that cargo release from nanoparticles could also variate depending on the resuspension fluid used. In this sense, using phosphate-buffered saline (PBS) at different pHs, pDNA release rates of up to 40% at 4 °C and 70% at 37 °C were achieved in the first 24 h (Supplementary Figure S5A,B, respectively). These results could also be explained by electrostatic interactions since PBS contains salts that create a different ionic environment from that of water, which could change the interplay between pDNA and PLGA. Indeed, these data are consistent with the literature, in which other authors have reported up to 50% pDNA release from PLGA nanoparticles diluted in PBS at pHs ranging from 4.5 to 7 during the first hours after synthesis [45,46]. 

Finally, for a given synthesis method, modification of the surfactant used can help to facilitate the cargo release [42]; however, other physicochemical characteristics of the NPs, such as particle size, would also vary. 

Strikingly, after 72 h of incubation in Milli-Q water at 37 °C, a sharp decrease in the released pDNA concentration measured was observed, which was compatible with the pDNA cleavage observed in the agarose gel electrophoresis (Supplementary Figure S6). Because this event was repetitively observed across several syntheses performed with different reagent stocks autoclaved and/or sterilized, and also because direct incubation of pDNA stock in water at 37 °C did not show plasmid degradation (Supplementary Figure S6, lane 1), this phenomenon is unlikely due to a DNase contamination of reagents and/or buffers. However, a depurination of the DNA helix has been reported in solutions containing primary amines [72], such as the PLGA-NH_2_ polymer, which might be exacerbated in our experimental conditions at 37 °C. Therefore, we cannot rule out that this nucleotide cleavage underlies the low plasmid DNA release measurable. 

Indeed, this hypothesis is in agreement with the results obtained in the release assay using PLGA NPs diluted in PBS at different pHs, where plasmid degradation, albeit at one week incubation times, can also be observed at 37 °C at 5.5 and 6 pH (Supplementary Figure S5D) but not at 4 °C (Supplementary Figure S5C). According to the literature, depurination is pH-dependent; the more acidic, the more depurination [73]. In addition, high temperature (37 °C) favors PLGA degradation into lactic and co-glycolic acids, which progressively acidify the medium [74,75].

Taken together, these data are compatible with a progressive acidification of the NP surrounding media due to PLGA hydrolysis boosted by high-temperature incubation at 37 °C. Acid pHs above 3 are known to promote depurination of DNA chains in a primary amine-rich environment such as amino-terminated PLGA, and thus explain the degradation observed in the agarose gels. Because PBS is a better pH buffering solution as compared to water, the acidification process is likely slowed, resulting in a later pDNA degradation (one week incubation vs. 72 h). 

**Figure 6:**
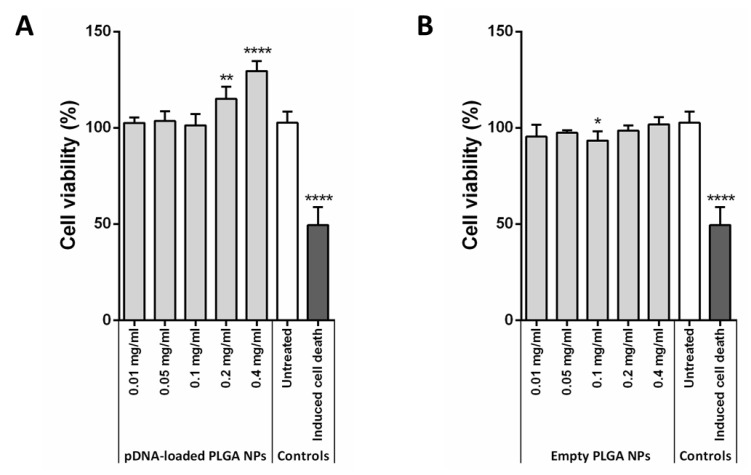
**In vitro cytotoxicity of pDNA-PLGA and PLGA nanoparticles**. Effects of pDNA-loaded (**A**) and empty (**B**) PLGA NPs synthesized by nanoprecipitation with amino-terminated PLGA on viability of NSC-34 cell line. * *p* < 0.05, ** *p* < 0.01, **** *p* < 0.0001, differences are calculated as compared to untreated cells.

Interaction between pDNA-loaded PLGA NPs and cells was also analyzed in terms of in vitro cell viability and uptake. Both empty and pDNA-loaded nanoparticles proved to be non-cytotoxic for NSC-34 cells (Figure 6). This result supports the literature indicating high biocompatibility and low toxicity of PLGA NPs [9]. 

Of note, at concentrations higher than 0.2 mg/mL, an increase in cell viability was observed (Figure 6A), which is possibly related to the plasmid activation of cell growth pathways, since empty NP treatment did not show any effect (Figure 6B). Alternatively, an induced increase in metabolic activity of the pDNA-NP-treated cells may result in a higher resazurin reduction at these concentrations. Further investigation would be necessary to understand the molecular mechanisms underlying these results.

**Figure 7:**
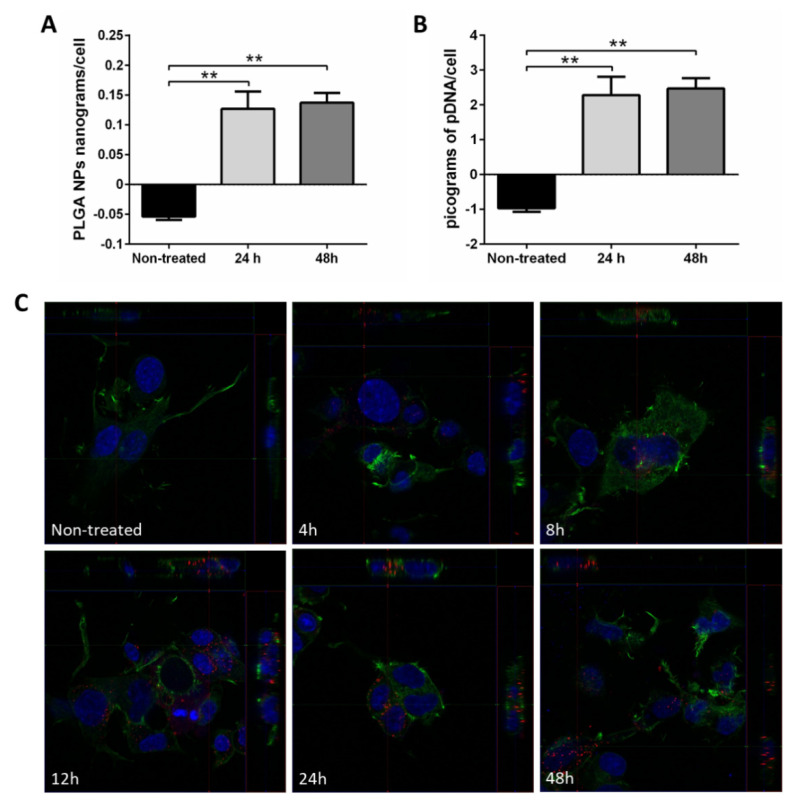
**In vitro uptake of pRFP-PLGA NPs synthesized by nanoprecipitation with amino-terminated PLGA by NSC-34 cells**. (**A**) Nanograms of PLGA NPs per cell. (**B**) Picograms of plasmid DNA per cell inferred from the amount of pDNA loaded per mg of PLGA NPs. (**C**) pDNA-loaded PLGA NPS cell uptake and localization of PLGA nanoparticles in the neuronal line NSC-34 at 4, 8, 12, 24 and 48 h. Actin filaments can be found in green, the nuclei in blue and pDNA-loaded PLGA NPs in red. * *p* < 0.05, ** *p* < 0.01, differences are calculated as compared to untreated cells.

Average single-cell PLGA and pDNA internalization were quantified by fluorimetry at 24 h and 48 h post treatment. Results showed that most pDNA-loaded PLGA NPs are internalized within the first 24 h (Figure 7A,B). Specifically, 0.127 ± 0.029 and 0.137 ± 0.016 PLGA nanograms per cell were internalized at 24 and 48 h, respectively. Based on the pDNA encapsulation efficiency, this corresponds to 2.282 and 2.469 picograms of pDNA per cell, which represent more than 235,000 copies of the plasmid per cell.

pDNA-loaded PLGA NPs internalization was also demonstrated using confocal microscopy (Figure 7C). Within 4 h of treatment, some small aggregates of nanoparticles on the surface of NSC-34 cell membrane and cytoplasm were already visible. At 8 h, nanoparticle uptake is higher and surface nanoparticles were rarely observed. The greatest number of cytoplasmic nanoparticles was reached at 12, 24 and 48 h after treatment. Furthermore, both quiescent and dividing cells were shown to internalize nanoparticles (Figure 7C, 8 h).

Unfortunately, no plasmid expression was observed based on RFP protein detection (Supplementary Figure S7), which may be a consequence of the slow plasmid release from PLGA observed for these nanoparticles in the aqueous environment. Nevertheless, pDNA uptake and expression is highly dependent on the cell line, and neuronal cellular models are often relatively difficult to internalize, even with commercial reagents such as lipofectamine. Alternatively, other aspects should be considered to achieve plasmid expression, namely increasing NP loading capacity or improving functional plasmid structure preservation and cell internalization. In this sense, pDNA PLGA NP cell internalization largely depends on particle size and zeta potential, clathrin-dependent and -independent endocytosis being the main pathways for particles between 90 and 120 nm [76]. As mentioned above, pDNA-loaded PLGA NPs have a negative zeta potential (even though PLGA-NH_2_ is cationic); however, a positive surface charge would allow cell membrane interactions and favor NPs internalization. Alternatively, cationic polymer coating and/or ligand conjugation might facilitate such interactions and cell uptake, but pDNA structural integrity and function should be re-evaluated in these conditions.

## 4. Conclusions

All three synthesis techniques evaluated in this work have been demonstrated to partially or totally alter the structure of large-size plasmid DNA.

In double emulsion, mechanical shear stress induced by sonicators and homogenizers is likely behind the nucleotide sequence disruption or the changes in the supercoiled functional conformation of the plasmid into open circular or linear isoforms. Moreover, for the first time, this work has assayed pDNA encapsulation using microfluidic systems where mechanical stress is milder and restricted to the flux of pDNA solution through the microchannels. However, the balance required between high shear stress to promote the emulsification process reaching high cargo loading and the low shear stress to prevent plasmid damage was not affordable under the conditions studied.

Alternatively, nanoprecipitation with amino-terminated PLGA resulted in high (57%) encapsulation efficiency and partial preservation of the pDNA supercoiled structure. However, pDNA cargo release in water is reduced likely due to strong electrostatic interactions between the PLGA and the pDNA, limiting its use for in vitro and in vivo approaches. Provided that this last drawback is successfully overcome, nanoprecipitation-synthesized nanoparticles manufactured in this work are promising candidates as large-size plasmid carriers, based on their size, encapsulation efficiency and conservation of pDNA supercoiled conformation. 

Furthermore, and although a significant cell uptake of these nanoparticles was demonstrated, encapsulation of this large-size pDNA resulted in negatively charged nanoparticles, which might hinder a high cell membrane interaction and uptake. Strategies such as ligand conjugation or cationic polymer coating of the nanoparticle (i.e., using polylysine) can be used to revert this charge and promote cell internalization. However, consequent modification of particle size or plasmid conformation should be considered. 

It is important to note that there are many and technically demanding variables and vector requirements that may affect cell transfection, i.e., nanoparticle size, cargo integrity preservation and release, surface charge or loading capacity. These parameters are often difficult to accomplish as a whole, as observed in this study with large plasmids, making most traditional nanoparticle synthesis methods not suitable to encapsulate sequences encoding gene editing machinery.

All in all, our work has compared the most commonly reported methodologies in the literature to encapsulate drugs and nucleic acids in PLGA polymer-based nanoparticles, including a new microfluidic emulsion system. However, the particularities of the plasmids used in gene editing technology in terms of size and charge make them sensitive to some of the processes and the most commonly used reagents. Further optimization or alternative synthesis methods are thus currently needed to adapt PLGA nanoparticles as delivery vectors for gene editing therapeutic technology. 

## Figures and Tables

**Table 1:** Properties of the pDNA-loaded PLGA NPs synthesized by double emulsion assisted with either ultrasound or microfluidics procedures and nanoprecipitation. NP formation efficiency, TEM diameter, zeta potential, percentage of encapsulation as well as stability of the encapsulated pDNA were evaluated. Data represent the mean ± SD (standard deviation) of at least two syntheses for each condition. NOC and SCI stand for nicked open circular isoform and supercoiled DNA index, respectively. * PEG interfered with the quantification of pDNA concentration, so percentage of encapsulation could not be determined.

Synthesis Method/Parameter Analysed	Ultrasound Assisted *W*/*O*/*W*		Microfluidics-Assisted *W*/*O*/*W*		Nanoprecipitation	
	Standard	Modified	Sodium Cholate	PVA	PLGA-COOH	PLGA-NH_2_
NP synthesis yield (%)	42.72 ± 5.28%		27.20 ± 10.68%	37.66 ± 11.74%	54.96%	28.48 ± 10.57%
TEM Diameter (nm)	116 ± 24	123 ± 67	212 ± 87	258 ± 140	88 ± 21	93 ± 29
Zeta Potential (mV)	−35.80 ± 4.36	−57.32 ± 10.16	−33.73 ± 2.91	−35.18 ± 12.93	−33.14 ± 1.91	−46.22 ± 12.49
Encapsulation efficiency %	33.16 ± 20.81%	Non-determined *	24.90%	50.91 ± 23.27%	0%	56.84 ± 16.09%
pDNA stability	Completely degraded (100%)	73.64% degraded, 8.80% supercoiled, 17.86% NOC	64.80% lineal, 35.20% NOC	45.01% lineal, 54.99% NOC	Not applicable	41.97% supercoiled, 58.03% relaxed
SCI	0%	0%	0%	0%	Not applicable	41.97% ± 8.06%

## Data Availability

All the data generated in this study are available within the article and Supplementary Materials.

## Supplementary Materials

The following are available online at https://www.mdpi.com/article/10.3390/nano11102723/s1, Figure S1: Schematic representation of agarose gel electrophoresis of pDNA, Figure S2: Correlation between zeta potential and pDNA encapsulation efficiency, Figure S3: Proof of concept for the agent triggering pDNA degradation in the double emulsion synthesis method assisted with batch-ultrasound procedure, Figure S4: Proof of concept for the agent triggering pDNA degradation in nanoprecipitation procedure, Figure S5: In vitro DNA release profile from nanoparticles synthetized by nanoprecipitation with amino-terminated PLGA. Figure S6: Agarose gel electrophoresis for supernatant obtained from pDNA-loaded PLGA nanoparticles synthesized by nanoprecipitation assisted by batch-magnetically mixed procedure and with amino-terminated polymer incubated at 37 °C, Figure S7: NSC-34 cells transfection with pRFP-PLGA NPs.
