# Supplementary material for: Encapsulation of Large-Size Plasmids in PLGA Nanoparticles for Gene Editing: Comparison of Three Different Synthesis Methods
Source: Nanomaterials (Basel). 2021 Oct 15;11(10):2723. doi: 10.3390/nano11102723 (PMC8541650; doi:10.3390/nano11102723)
Supplement: Supplementary file 1 [file nanomaterials-11-02723-s001.zip › nanomaterials-1377059-supplementary-conversion.pdf]

nanomaterials

MDPI

Supplementary material

Encapsulation of Large-Size Plasmids in PLGA Nanoparticles for Gene Editing: Comparison of Three Different Synthesis Methods

Tresa López-Royo [1,2,3,4], Víctor Sebastián [4,5,6,7], Laura Moreno-Martínez [1,2,3,4], Laura Uson [4,5,6,7], Cristina Yus [5,6], Teresa Alejo [4,5,6,7], Pilar Zaragoza [1,2,3,4], Rosario Osta [1,2,3,4], Manuel Arruebo [4,5,6,7] and Raquel Manzano [1,2,3,4,*]

[1] Department of Anatomy, Embryology and Animal Genetics, University of Zaragoza, Calle Miguel Servet 177, 50013 Zaragoza, Spain;
[2] Centro de Investigación Biomédica en Red de Enfermedades Neurodegenerativas (CIBERNED), 28031 Madrid, Spain;
[3] Agroalimentary Institute of Aragon (IA2), Calle Miguel Servet 177, 50013 Zaragoza, Spain;
[4] Aragón Health Research Institute (IIS Aragón), 50009 Zaragoza, Spain;
[5] Instituto de Nanociencia y Materiales de Aragón (INMA), CSIC-Universidad de Zaragoza, 50009 Zaragoza, Spain;
[6] Department of Chemical Engineering, University of Zaragoza, Campus Río Ebro—Edificio I+D, C/Poeta Mariano Esquillor S/N, 50018 Zaragoza, Spain;
[7] Networking Research Center on Bioengineering, Biomaterials and Nanomedicine, CIBER-BBN, 28029 Madrid, Spain.
* Correspondence: rmanzano@unizar.es

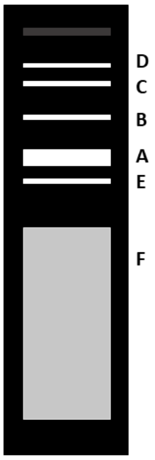

Figure S1. Schematic representation of agarose gel electrophoresis of pDNA. (A) Supercoiled DNA, (B) linear DNA, (C) relaxed DNA, (D) nicked open-circular DNA, (E) denatured supercoiled DNA, (F) degraded DNA.

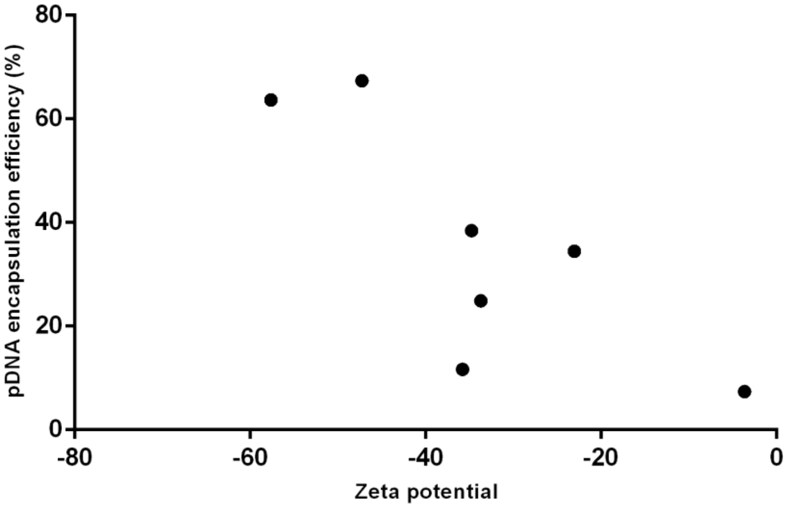

Figure S2. Correlation between zeta potential and pDNA encapsulation efficiency. Each point on the graph represents a synthesis performed. All synthesis replicates are included in this graph regardless of the synthesis method drived by, except for nanoprecipitation with PLGA-COOH and modified ultrasound-assisted double emulsion replicates, as they did not show pDNA entrapment or the percentage of encapsulation could not be determined, respectively.

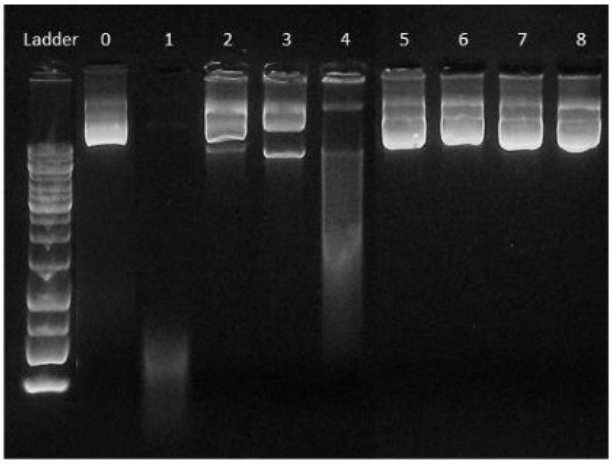

Figure S3. Proof of concept for the agent triggering pDNA degradation in the double emulsion synthesis method assisted with batch-ultrasound procedure. Image shows the migration and structural integrity of pRFP when exposed to (1) sonication for 60s at 40% amplitude, (2) homogenizer for 60s at low speed, (3) homogenizer for 60s at medium speed, (4) homogenizer for 60s at high speed, (5) DCM + 0.01% sodium cholate, (6) DCM + 0.1% sodium cholate, (7) DCM + 0.6% sodium cholate, (8) DCM + 2% sodium cholate. Samples were run next to the pRFP stock as control (0).

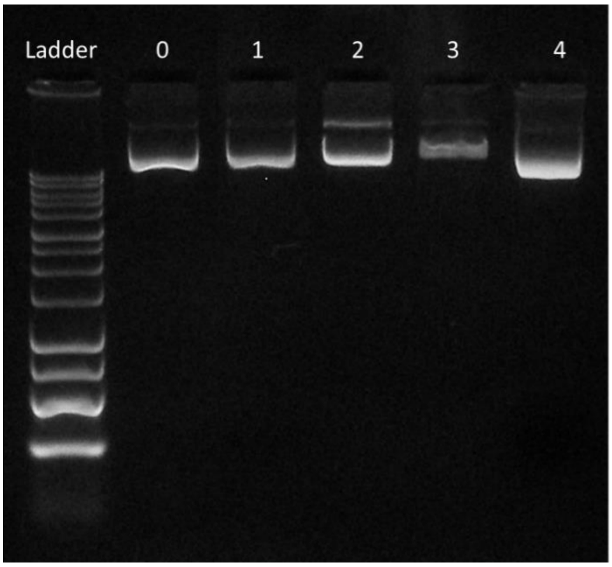

Figure S4. Proof of concept for the agent triggering pDNA degradation in nanoprecipitation procedure. Image shows the migration and structural integrity of pRFP when subjected to (1) passage through a syringe, (2) pluronic F-127 for 5 hours, (3) DMF for 5 hours, (4) THF for 5 hours. Samples were run next to the pRFP stock as control (0).

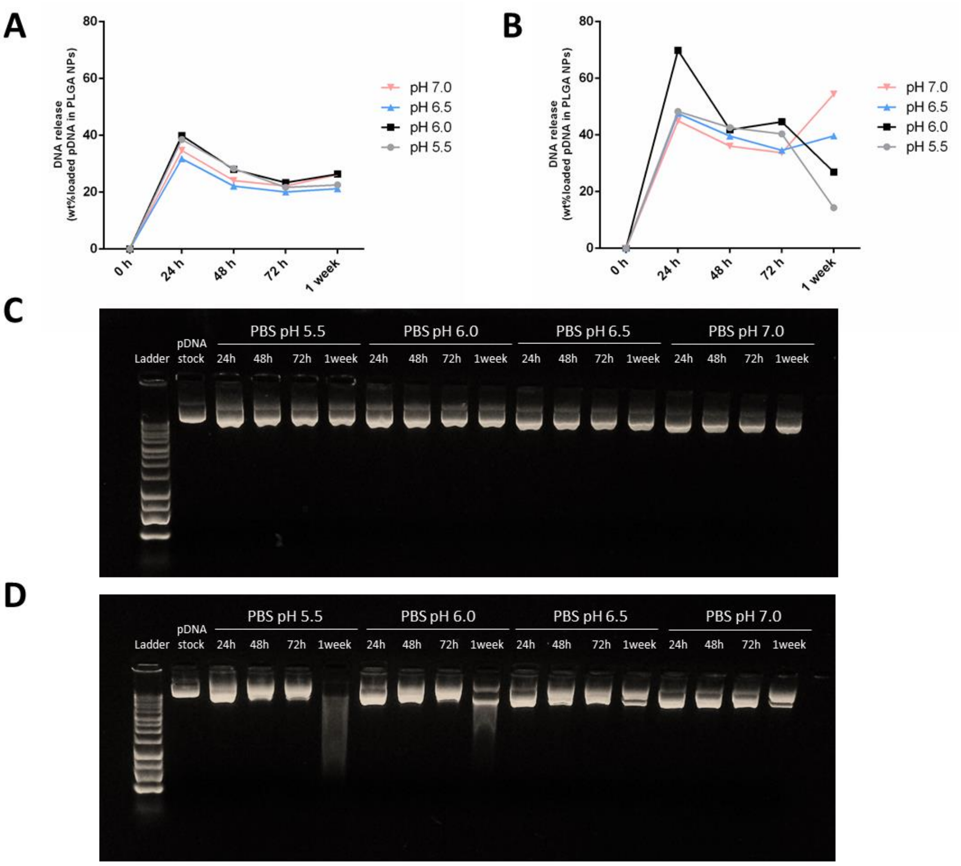

Figure S5. In *vitro* DNA release profile from nanoparticles synthetized by nanoprecipitation with amino-terminated PLGA. (A) DNA release in PBS at 4°C (B) DNA release in PBS at 37°C (C) Migration and structural integrity of DNA released at 4 °C (D) Migration and structural integrity of DNA released at 37°C. % released = [mass DNA released into solution]/[initial total mass DNA encapsulated].

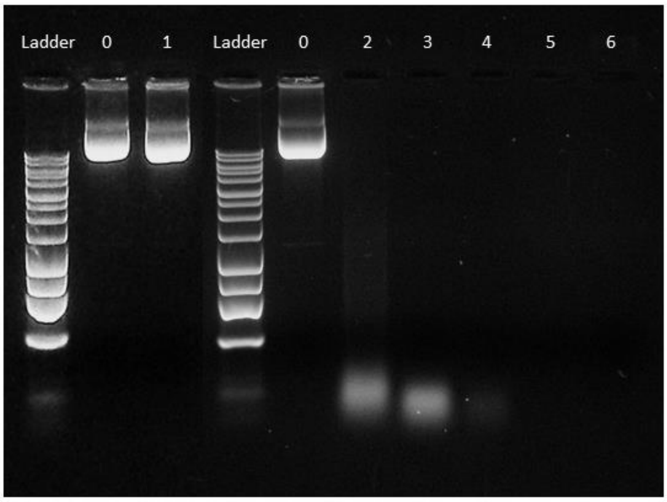

Figure S6. Agarose gel electrophoresis for supernatant obtained from pDNA-loaded PLGA nanoparticles synthesized by nanoprecipitation assisted by batch-magnetically mixed procedure and with amino-terminated polymer incubated at 37°C. Image shows the migration and structural

integrity of pRFP plasmid stock incubated at 4°C (lane 0) and 37°C (lane 1) for 24h and pRFP released at 37°C for24h (lane 2), 48h (lane 3), 72h (lane 4), 1 week (lane 5) or 2 weeks (lane 6).

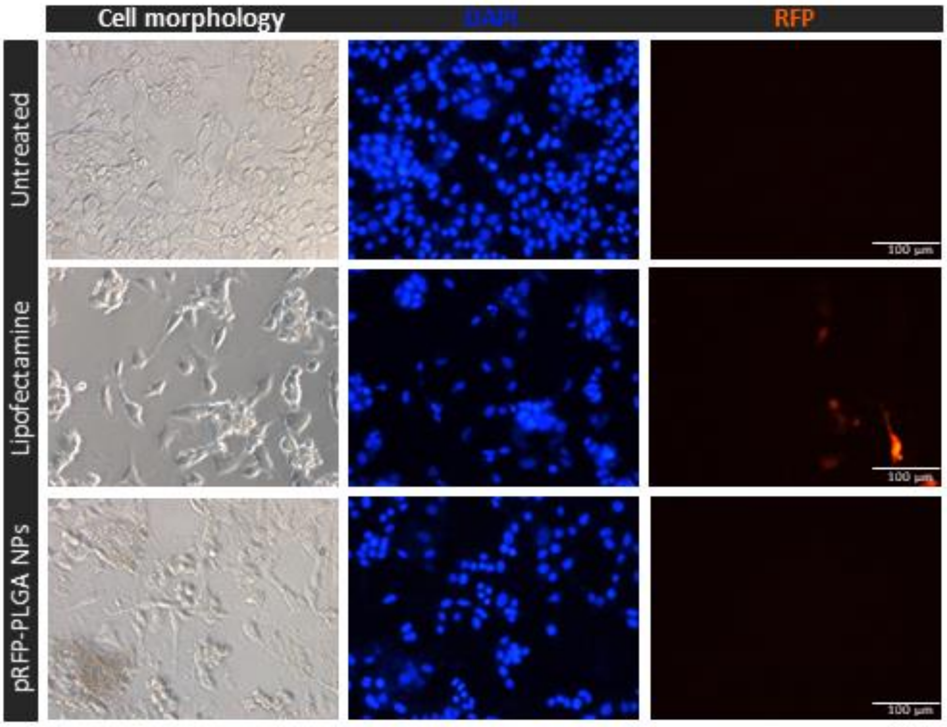

Figure S7. NSC-34 cells transfection with pRFP-PLGA NPs. NSC-34 cells were grown in 24-well plates (100,000 cells/well), transfected with the amount of nanoparticles corresponding to 2 µg of encapsulated plasmid and incubated for 72 hours (24 h uptake + 48 h expression). pRFP plasmid used for this experiment encoded for the red fluorescent protein (RFP), whose expression was used to indicate correct transfection. On the other hand, DAPI dye was used to stain the nuclei of cells. Untreated cells and cells transfected with lipofectamine and 2 µg of pRFP, with an equal expression period of 48 h, were included as a negative and positive control, respectively.
